# The prevalence of 30‐day readmission after acute myocardial infarction: A systematic review and meta‐analysis

**DOI:** 10.1002/clc.23238

**Published:** 2019-08-12

**Authors:** Huijie Wang, Ting Zhao, Xiaoliang Wei, Huifang Lu, Xiufang Lin

**Affiliations:** ^1^ Department of Cardiology and Cardiovascular Intervention, Interventional Medical Center The Fifth Affiliated Hospital of Sun Yat‐sen University Zhuhai PR China

**Keywords:** acute myocardial infarction (AMI), meta‐analysis, prevalence, readmission

## Abstract

**Objective:**

The 30‐day readmission is associated with increased medical costs, which has become an important quality metric in several medical institutions. This current study is aimed at clarifying the prevalence, the underlying risk factors, and reasons of the 30‐day readmission after acute myocardial infarction (AMI).

**Methods:**

PubMed, Cochrane Library, and EMBASE were systematically searched to identify eligible studies. Random‐effect models were employed to perform pooled analyses. Means and 95% confidence intervals (CIs) were used to estimate prevalence and reasons for 30‐day readmission. We also used Odds ratios (ORs) to explore the potential significant predictors of risk factors of 30‐day readmission after AMI. Potential publication bias was assessed using funnel plot and Begg'test.

**Results:**

A total of 14 relevant studies were included in this systematic review and meta‐analysis. The pooled 30‐day readmission rate of AMI was 12% (95% CI 0.11‐0.14). Acute coronary syndrome (ACS), angina and acute ischemic heart disease, and heart failure (HF) were the principal cardiovascular reasons of 30‐day readmission. Meanwhile, non‐specific chest pain was regarded as the significant cause among non‐cardiovascular reasons. The common co‐morbidities kidney disease, HF and diabetes mellitus were significant risk factors for 30‐day readmission. No significant publication bias was found by funnel plot and statistical tests.

**Conclusions:**

The 30‐day readmission rate of post‐AMI ranged from 11% to 14% and can be mainly attributed to cardiovascular and non‐cardiovascular events. The common co‐morbidities, such as kidney disease, HF, and diabetes mellitus were significant risk factors for 30‐day readmission.

**Abbreviations**
ACS: acute coronary syndrome
AMI: acute myocardial infarction
COPD: chronic obstructive pulmonary disease
CMS: Centers for Medicare and Medicaid Services
HF: heart failure
HRRP: Hospital Readmission Reduction Program
MI: myocardial infarction
NSTEMI: non‐ST‐segment elevation myocardial infarction
NRD: Nationwide Readmissions Database
ORs: odds ratios
STEMI: ST‐elevation myocardial infarction
UK: United Kingdom

## 1 INTRODUCTION

Acute myocardial infarction (AMI), one of the most serious coronary artery diseases, is associated with increased morbidity and mortality, impaired quality of life.^1^ Approximately, one‐sixth patients who was diagnosed AMI would have unplanned readmission during 30 days of hospital discharge, which estimated direct costs of $1 billion of annual Medicare expenditures in the United States.^2^ Statistically, nearly 20% of Medicare beneficiaries was readmitted within 30‐day after AMI.^3^ Therefore, reducing the rates of rehospitalization has attracted attention from policymakers and medical workers as a way improve the quality of care and reduce costs, payment incentives and Medicare hospital readmission penalties were created to reduce readmission rates.^4^, ^5^ Beginning in year 2013, the Hospital Readmission Reduction Program (HRRP) was carried out by the US Centers for Medicare& Medicaid Services (CMS) to improve financial incentives for decreasing readmission, which hospitals received penalizing according to higher‐than‐expected risk‐standardized 30‐day readmission rates for heart failure, myocardial infarction, and pneumonia.^5^

Re‐hospitalization is a frequent negative outcome for both hospitals and patients, and is an enormous economic burden to the Medicare beneficiaries and private payer.^3^ There was a weak but significant correlation between the reduction of the 30‐day readmission and 30‐day hospital mortality after hospital discharge.^6^ The re‐admission rate of 30‐days after myocardial infarction reduced from 20.5% to 15.8% from 2001 to 2003 to 2009 to 2011, but this trend slightly decreased after adjusting patient characteristics and treatment methods.^7^ Predicting risk factors and reasons of 30‐day readmission after AMI could help clinicians to actively identify patients with the highest possibility to benefit from intensity of the readmission intervention, so as to optimize limited medical resources allocation and implement beneficial and sustainable intervention.^8^, ^9^ However, many previous studies were performed in single‐center study with a small sample size and had shown inconsistent results for readmissions after 30‐days of AMI. Hence，it is necessary to further understand the prevalence, potential causes, and risk factors for readmission.

Therefore, the purpose of our study was aimed at clarifying the prevalence, identify, and compare the potential risk factors and reasons for AMI of the 30‐day readmission. Moreover, we discuss the potential intervention strategies to reduce the identified risk factors and causes of readmission.

## 2 METHODS

This present systemic review was performed according to pre‐designed protocol, which was conducted under the Meta‐analysis of Observational Studies in Epidemiology.^10^

### 2.1 Search strategy and study selection

Relevant literatures were systematically acquired from three electronic databases using the PubMed, EMBASE, and Cochrane Library (contained Cochrane Central Register of Controlled Trials, Cochrane Database of Systematic Reviews, and the Database of Abstracts of Reviews of Effect) for study prevalence of 30‐day readmission after AMI. The articles published date from inception to May 26, 2019 to obtain any possible inclusion. The two reviewers independently performed title words to search eligible articles that included following two concepts: (a) readmission (readmission*, re‐admission*, rehospitalization*, re‐hospitalization*, reattendance*, re‐attendance*, readmittance*, re‐admittance*), and (b) acute myocardial infarction (myocardial infarction, MI, acute myocardial infarction, AMI, non‐ST‐segment elevation myocardial infarction, NSTEMI, ST‐elevation myocardial infarction, STEMI). A total of 1104 studies were identified. The detailed search strategies were provided in the Appendix S1.

### 2.2 Study inclusion and exclusion criteria

This systematic review and meta‐analysis of inclusion criteria was directed on the basis of the Preferred Reporting Items for Systematic Review and Meta‐analysis protocols (PRISMA‐P). Articles acquired from three electronic databases were combined and duplicate literatures were removed. Pooled studies by the title and abstract were screened and removed in the light of systematic review, meta‐analysis, commentary, irrelevant title, not involving patients of AMI, no related to 30‐day readmission, and no concerned to risk factors or causes of readmission. Afterwards, the rest of the articles were removed by browsing their full‐text according to following exclusionary criteria including

1. Conference abstract or commentary;
2. Not reporting risk factors or reasons of hospital readmitted;
3. Not regarding 30‐day readmission rate or data;
4. Studies did not provide available data;
5. Data derived from subset of the study patients or provide data not available.

**Figure 1:**
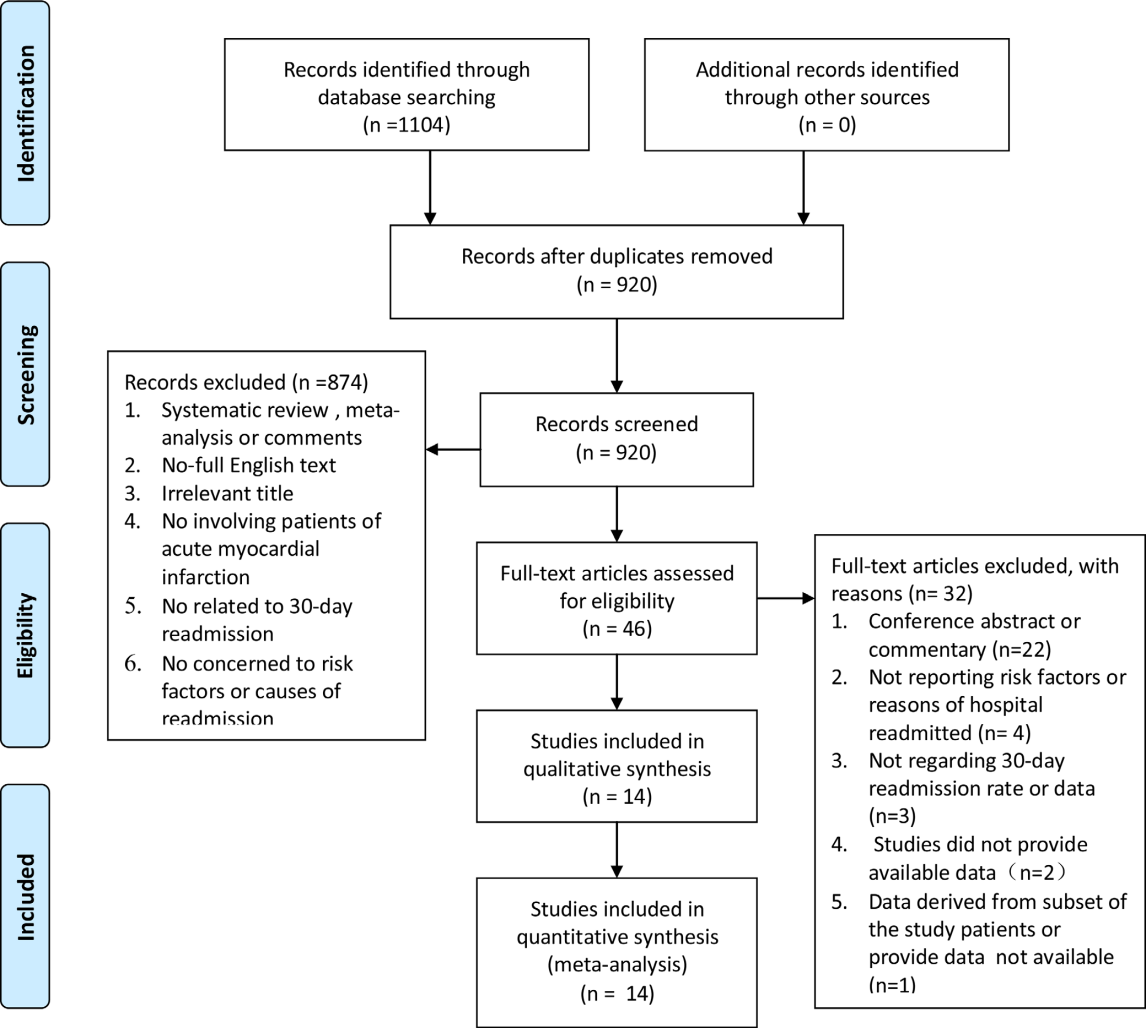
Flow diagram of studies selection

Details inclusion and exclusion criteria of selected studies were presented in Figure 1.

### 2.3 Data extraction and methodological quality assessment

The standard EXCEL forms were used to extracted relevant data including study period, diagnoses, country, data source, study population, demographic characteristics, simple size, prevalence, definition of 30 day readmission, underlying risk factors as well as causes of 30‐day readmission. Using multivariable analysis of risk factors and reasons of readmission identified in two or more studies was collected. The quality of included studies was assessed using standard from Critical Appraisal of the Health Research Literature: Prevalence or Incidence of a Health Problem,^11^ which including eight items (ie, sample size, sample design, sampling frame, study and setting, measures, unbiased assessors, response rate and refusers, and prevalence rates) and one point for each item. The quality of individual studies was classified low‐quality when total score is less than 6 or highly‐quality if whole score is in 6 or more scores.

### 2.4 Statistical analysis

The primary outcome is the prevalence of 30‐day readmission and the secondary outcomes include underlying causes and risk factors for 30‐day readmission after AMI. Random‐effect models were employed to perform pooled analyses because clinical heterogeneity across contained studies. Means and 95% confidence intervals (CIs) were used to estimate prevalence and reasons for 30‐day readmission. We conducted subgroup analyses by stratifying of region, study population, quality of included studies find out the sources of heterogeneity. We also used odds ratios (ORs) to explore the potential significant predictors of risk factors of 30‐day readmission after AMI. Potential publication bias was assessed using funnel plot and Begg'test. The two articles^12^, ^13^ of data source acquired from Nationwide Readmissions Database (NRD) which is a large national database. Using influence analysis was performed to explore whether the overlap study population would influence the overall pooled readmission rate. If the *P*‐value is less than .05, we consider the correlation to be statistically significant. All meta‐analysis was performed using State 12.

## 3 RESULTS

### 3.1 Search results and study characteristics

The overall of 1104 articles were identified in this meta‐analysis after systematically searching, which used PubMed, Cochrane Library, and EMBASE result in 138, 697, and 269 entries, respectively. The 920 unique records after eliminating 184 duplication literatures would be further removed in a stepwise by the title, abstract, full‐text according to pre‐specified study inclusion and exclusion criteria. Ultimately, 14 observational studies^7^, ^12^, ^13^, ^14^, ^15^, ^16^, ^17^, ^18^, ^19^, ^20^, ^21^, ^22^, ^23^, ^36^ met the eligibility criteria and were comprised in this systematic review, which the detailed filtering process was presented in Figure 1. Ten of the 14 studies were performed in the America, which the rest of individual studies were conducted Chain, France, Spain, and the United Kingdom (UK). Eleven articles included patients with AMI, rest of solely one involved people with NSTEMI and other two assessed STEMI patients. The sample size of each included study was different. We divided the sample size into three layers according to sample size less than 1000, 1000‐10 000, and more than 10 000. Six studies included all patients, six articles included adults (aged≥18 years), and only one article included middle‐aged crowd (aged 18‐64 years), or elderly patients (aged ≥65 years). Four articles of population were defined as unplanned readmission and other were defined as readmission which was no strict distinction between planned readmission and unplanned readmission in the original study. Details baseline characteristics of selected studies were presented in Table 1. Three studies were identified as low‐quality and another 11 as high‐quality (Appendix S2).

**Table 1:** Characteristics of included studies

Author (year)	Country	Study period	Readmission/total patients	Diagnoses	Data source	Study population	Definition of 30 day readmission
Li et al^36^	China	2012.12‐2014.5	215/3387	AMI	53 acute‐care hospitals	Adults (aged ≥18 years)	Unplanned
Zabawa et al^23^	France	2011‐2013	137/624	AMI	EGB database	Elderly (aged ≥65 years)	Readmission
Nguyen et al^15^	America	2009‐2010	107/826	AMI	Electronic health record data from 6 hospitals in north Texas	Adults (aged≥18 years old)	Unplanned
Kim et al^12^	America	2010–2014	87 415/709548	STEMI	NRD	All patients	Readmission
Rodriguez‐Padial et al^16^	Spain	2007‐2013	1811/33538	AMI	MBDS of SNHS	All patients	Readmission
Kwok et al^17^	UK	2012‐2014	171/1869	AMI	MINAP	All patients	Unplanned
Khera et al^13^	America	2013.1‐2013.12	69 517/478247	AMI	NRD	Adults (aged ≥18 years)	Readmission
Tisminetzky et al^18^	America	1999‐2009	335/2249	NSTEMI	Worcester Heart Attack Study	All patients	Readmission
Dreyer et al^19^	America	2007‐2009	4775/42518	AMI	Healthcare Cost and Utilization Project‐State Inpatient Database	Middle‐aged crowd (aged 18–64 years)	Readmission
Chen et al^7^	America	2001‐2011	890/4810	AMI	3 central Massachusetts hospitals	Adult residents	Readmission
Ranasinghe et al^21^	America	2007–2009	16 117/107256	AMI	All‐payer administrative dataset from California	Adults (aged ≥18 years)	Unplanned
Ben‐Assa et al^20^	America	2009‐2012	52/897	AMI	“SHL”‐Telemedicine electronic database	Adults (aged 23‐90 years)	Readmission
Brown et al^22^	America	2006‐2011	127/1271	STEMI	Dartmouth‐Hitchcock Medical Center cardiac catheterization laboratory	All patients	Readmission
Dunlay et al^14^	America	1987‐2010	561/3010	AMI	Olmsted County residents	All patients	Readmission

Abbreviations: AMI, Acute myocardial infarction; EGB, Échantillon Généraliste de Bénéficiaires; MINAP, Myocardial Infarction National Audit Project; MBDS, Minimum Basic Data Set; NRD, Nationwide Readmissions Database; NSTEMI, non‐ST‐segment elevation myocardial infarction; SNHS, Spain's National Health System; UK, United Kingdom.

### 3.2 Thirty‐day readmissions rate

**Figure 2:**
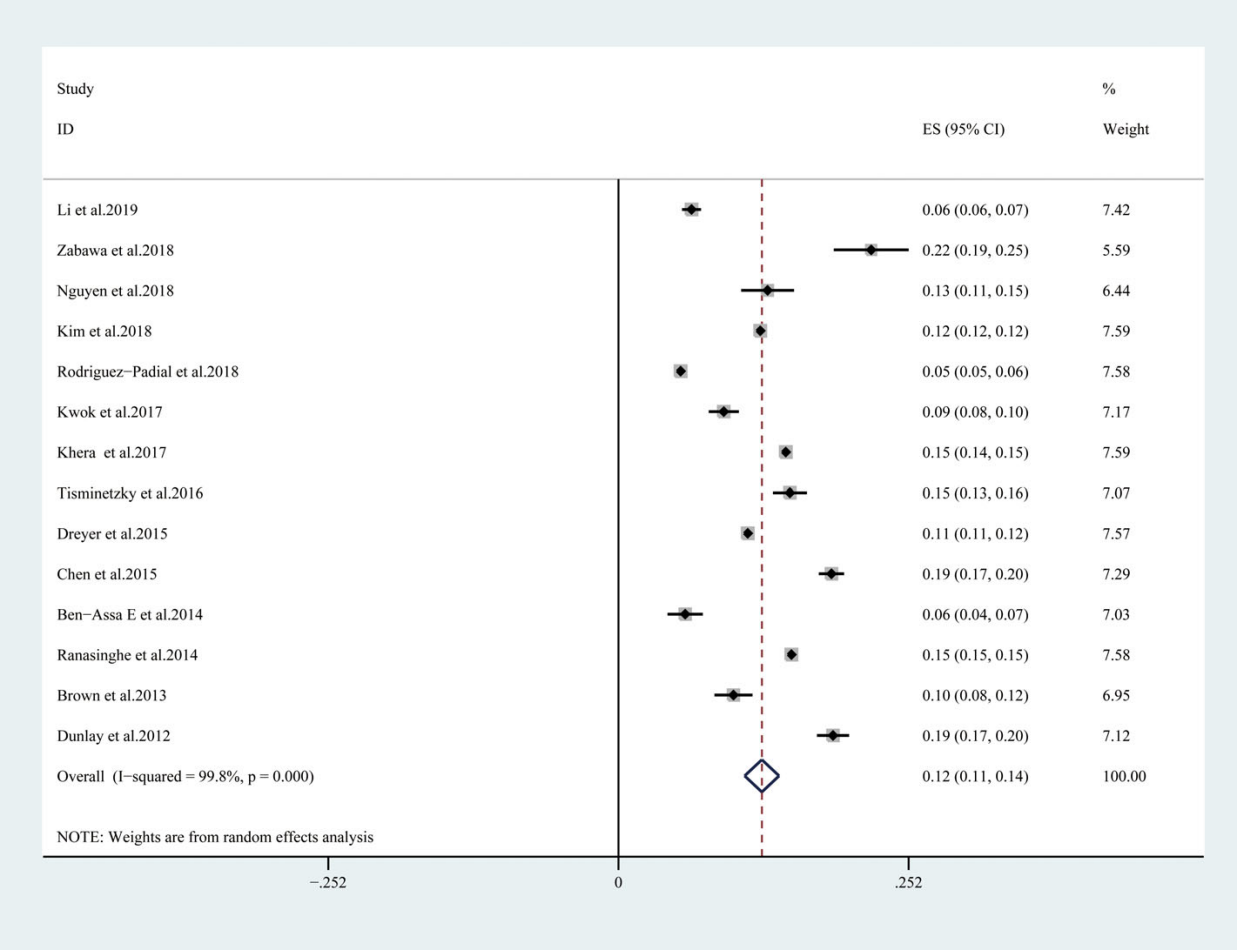
Forest plot of 30‐day readmission rate after acute myocardial infarction

In brief, the pooled prevalence of 30‐day readmission was 12% (95% CI 0.11‐0.14; Figure 2). Heterogeneity is extremely high (*I* ^2^ = 99.8, *P* = 0), which using funnel plot analysis show asymmetry (Appendix S3). Further subgroup analysis stratified by region, sample size, quality of included studies and definition of 30‐day readmission (Appendix S4). The rate of unplanned readmission or nor‐America region (unplanned readmission 11%, 95% CI, 0.06‐0.16, non‐America 10%，95% CI, 0.07‐0.13) was lower than readmission and America region (readmission, 13%, 95% CI, 0.11‐0.15，America 13%，95% CI, 0.12‐0.15) in 30 days after MI. The results of subgroup analyses based on quality of included studies and sample size were basically consistent with the overall pooled effect. The influence analysis found that the pooled prevalence of 30‐day readmissions after discharge fluctuated ranging from 10% to 15% (Appendix 5).

### 3.3 Reasons for readmission

This 32 causes of re‐hospitalization were classified two categories: (a) cardiovascular causes: cardiac cause was vitally important reasons associated with 30‐day readmission, which accounted for 58% of the all causes after AMI. Acute coronary syndrome (ACS), angina, and ischemic heart disease, heart failure (HF), HF/acute pulmonary edema, AMI, and chest pain were identified to be associated with an increased risk of 30‐day readmission. (b) Non‐cardiovascular causes: the non‐cardiac cause was the significantly associated with a higher risk of 30‐day readmission. The non‐specific chest pain appeared in three articles was most frequent reasons related to 30‐day re‐hospitalization, which accounted for 24% of the all causes after AMI. Some other causes, such as complications of care or procedural, respiratory disease, renal disorders, septicemia/shock, etc. leaded to 30‐day readmission after AMI. The causes of 30‐day readmission are shown in Table 2.

**Table 2:** Reasons for 30‐day readmission after acute myocardial infarction in included studies

Causes	No. of studies reporting individual cause	No. of individual‐cause readmissions	No. of all‐cause readmissions	Readmission rate with 95% CI	*I* ^2^ (%)	*P* for heterogeneity
Cardiovascular causes						
Cardiac causes	5	54 083	92 628	0.58 (0.54，0.62)	94.5	0
ACS	2	40	223	0.18 (0.12，0.23)	6.2	0.302
Angina and ischemic heart disease	7	17 523	109 472	0.18 (0.13，0.22)	99.6	0
HF	6	15 042	104 578	0.13 (0.11，0.16)	96.5	0
HF/acute pulmonary edema	2	630	4946	0.12 (0.09, 0.015)	57.6	0.124
AMI	6	12 008	109 301	0.10 (0.09，0.11)	90.7	0
Chest pain	4	1699	21 671	0.06 (0.02，0.11)	99.2	0
Arrhythmias and conduction disorders	7	4373	109 309	0.03 (0.03，0.04)	91.7	0
Cardio‐respiratory failure	2	531	20 892	0.02 (0.01，0.03)	94.2	0
Hypertension/HD	4	263	21 115	0.01(0.01，0.02)	96.8	0.005
Valvular/rheumatic heart disease	2	84	20 892	0.00(0.00，0.01)	93.1	0
Non‐cardiovascular causes						
Non‐cardiac causes	5	92 628	38 538	0.41 (0.37, 0.45)	94	0
Non‐specific chest pain	3	5654	87 638	0.24(0.06，0.41)	96.5	0
Complications of care or procedural	4	6936	108 949	0.06 (0.05，0.07)	94.1	0
Respiratory disease/septicemia	7	5977	109 309	0.06 (0.05，0.07)	92.2	0
Renal disorders	6	2539	109 257	0.03(0.01，0.04)	98.6	0
Septicemia/shock/spesis	3	842	21 063	0.03 (0.01，0.04)	95.6	0
Bleeding	4	2412	108 478	0.02 (0.02，0.03)	85.2	0
CVA or TIA or stroke	7	2302	109 387	0.02(0.02，0.03)	83.9	0
Other peripheral vascular disease	2	453	20 892	0.02 (0.02，0.02)	0.0	0.686
Diabetes and its complications	2	335	20 892	0.02 (0.01，0.02)	0.0	0.345
Fluid and electrolyte disorders	2	13	813	0.02(0.01，0.02)	0	0.575
UTI and urinary system	4	315	21 705	0.01 (0.01，0.02)	85.9	0
Pulmonary embolus	4	279	21 705	0.01 (0.01，0.01)	0	0.788
Dizziness, presyncope, syncope, fall	3	209	21 063	0.01 (0.00，0.01)	15.1	0.308
Clostridium difficile‐associated infection	2	155	20 892	0.01(0.00，0.01)	96.6	0
Anemia	3	154	21 063	0.01 (0.01，0.01)	30.4	0.238
Pleural effusion/pneumothorax/pleurisy	3	152	21 534	0.01 (0.01，0.01)	62.9	0.067
Cellulitis	2	119	20 892	0.01 (0.00，0.01)	28.8	0.236
Hip fracture	2	79	20 892	0.00 (0.00, 0.01)	96.8	0
Primary cancer	2	44	20 892	0.00 (0.00，0.00)	0	0.743
Generally unwell	2	23	813	0.00 (0.00，0.00)	0	0.743

Abbreviations: ACS, acute coronary syndrome; AMI, acute myocardium infarction; CVA, cerebrovascular accident; HD, congenital heart; HF, heart failure; TIA, transient ischemic attacks; UTI, urinary tract infection.

### 3.4 Risk factors for 30‐day readmission

Fifteen risk factors were identified in two or more studies with using multivariable analysis. This commonly disease of comorbidity, such as kidney disease (included renal failure, renal function (Cr > 2 mg/dL), acute kidney injury, end‐state renal disease/hemodialysis), diabetes mellitus, chronic obstructive pulmonary disease (COPD), HF, peripheral vascular disease, cardiac arrhythmia was closely related to increased 30‐day readmissions. Similarly, the uncommonly blood disease and fluid/electrolyte disorders were also recognized weaker factors of re‐hospitalization. The female sex involved five studies was correlation with increased readmissions. However, the hypertension, valvular or rheumatic heart disease, length of stay at index hospitalization, anterior MI, previous MI, and cardiogenic shock was not specifically correlation with 30‐day readmissions. The risk factors of 30‐day readmission are shown in Table 3.

**Table 3:** Significant risk factors for 30‐day readmission on multivariate analysis

Risk factors	Li et al^36^	Zabawa et al^23^	Nguyen et al^15^+	Kim et al^12^	Rodriguez‐Padial et al^16^	Kwok et al^17^	Khera et al^13^	Tisminetzky et al^18^	Dreyer et al^19^	Chen et al^7^	Ranasinghe et al^21^	Ben‐Assa E^20^	Brown et al^22^	Dunlay et al^14^	Pooled OR with 95%CI	I2(%)	P for heterogeneity
Female sex	—	—	NS	*	*	—	*	—	—	*	—	—	—	—	1.17(1.15 1.20)	0.0	0.594
Hypertension	—	—	—	*	—	—	—	NS	—	NS	—	—	—	—	1.03(0.93 1.15)	37.7	0.201
Diabetes mellitus	—	—	*	*	*	—	*	*	—	NS		—	—	—	1.23(1.18 1.28)	51	0.069
COPD	—	—	—	*	*	—	*	NS	—	NS	—	—	—	—	1.20(1.11 1.30)	82.1	0
HF	—	—	—	*	*	—	*	*	—	*	—	—	—	—	1.32(1.20 1.44)	91.8	0
Peripheral vascular disease	—	—	—	*	—	—	*	NS	—	NS	—	—	—	—	1.17(1.14 1.21)	0	0.766
Kidney disease	—	—	*	*	*	—	*	*	—	*	—	—	—	—	1.53(1.29 1.81)	88	0
Blood disease	—	—	—	*	*	—	*	—	—	—	—	—	—	—	1.16(1.09 1.23)	83	0.003
Fluid/electrolyte disorders	—	—	—	*	—	—	*	—	—	—	—	—	—	—	1.09(1.01 1.17)	88.6	0.003
Valvular or rheumatic heart disease	—	—	—	NS	*	—	*	**—**	—	—	—	—	—	—	1.12(0.92 1.36)	93.3	0
Length of stay at index hospitalization	—	—	—	—	—	—		*	—	*	—	—	—	—	1.21(0.81 1.82)	88.8	0.003
Cardiac arrhythmia	—	—	—	*	—	—	*	NS	—	*	—	—	—	—	1.14(1.08 1.20)	62.5	0.046
Anterior MI	—	—	—	—	*	—	*	—	—	—	—	—	—	—	2.26(0.54 9.45)	99.6	0
Previous MI	—	—	—	NS	*	—	*	—	—	—	—	—	—	—	1.22(0.98 1.52)	98	0
Cardiogenic shock	—	—	—	—	NS	—	—	—	—	*	—	—	—	—	1.24(0.81 1.91)	88	0.004

*Notes*: NS, no statistical significance;  + AMI READMITS score (first‐day model); COPD, chronic obstructive pulmonary disease; HF, heart failure; kidney disease included renal failure, renal function (Cr > 2 mg/dL),acute kidney injury, end state renal disease/hemodialysis; MI, myocardial infarction; blood system disease refers to iron deficiency anemia or other anemia other rather leukemia.

* Statistical signifcance (*P* < .05).

### 3.5 Publication bias

No significant publication bias was found by funnel plot and statistical test (Begg test, *P* = .274; Appendix 3). However, asymmetric funnel plots suggested potential publication bias in the current meta‐analysis.

## 4 DISCUSSION

To the best of our knowledge, this study is the initial systematic review and meta‐analysis of all reported assessed prevalence of 30‐day readmission after AMI. The reasons of 30‐day readmission were mainly attributed to cardiac factors and non‐cardiac factors. The non‐specific chest pain was deemed to most frequent all reasons of the non‐cardiac problems for readmission. Meanwhile, most causes of cardiac readmissions were due to angina and acute ischemic heart disease, ACS, HF, AMI, chest Pain, etc. In addition, kidney disease, female sex, diabetes mellitus, COPD, HF is the principal predictor of early readmission.

In this meta‐analysis, the pooled analysis contained 14 articles found that the 30‐day readmission rate after AMI was 12% (95% CI 11%‐14%). Previous studies have shown that the re‐admission rate of myocardial infarction within 30 days is 19.9%，in which nearly 67.6% of re‐admissions occur within 15 days after discharge.^24^ Subgroup analyses indicated that 30‐day readmission prevalence was more stable in America region and sample size >10 000 than those not America region and sample size <1000. It may be that multicenter and large sample studies ware generally carried out in the United States, and the specific reasons need to be further explored. The rate of readmission was higher than unplanned readmission of 30‐day readmission after AMI, which can be seen in the literature we included. Age, sex, data source, study period, diagnose, and study population were all different in the contain literatures and this probably reasons of heterogeneity. Because of inconsistent reporting and research data limitation, we were unable to conduct subgroup analysis on the all variables except for sample size, region, and quality of included studies. The influence analysis found that the pooled prevalence of 30‐day readmissions after discharge did not substantially fluctuate，so the population of the two overlapping studies did not affect the overall readmission rate.

The present study also suggested several causes resulting in 30‐day readmission after AMI including cardiovascular causes and non‐cardiovascular factors, which may offer possible right direction to decrease the 30‐day readmission rate. During the cardiac factors accounted for 58% varied from 54% to 62%, while non‐cardiac factors only had proportion of 41% ranged from 37% to 45% for 30‐day readmission rate. Non‐cardiac factors，the 24% of readmissions were attributable to non‐cardiac chest pain, which three articles made mention of non‐cardiac chest pain and its incidence fluctuated from 6% to 41%. Previous studies also indicated that non‐cardiac chest pain accounted for one‐third of the chest pain patients in AMI and was associated with disease‐specific quality of life and general physical and mental health impairment.^25^ Another some important reason associated with 30‐day readmission after AMI result from complications of care or procedural, respiratory disease, renal disorders, septicemia/shock.

We also identified that kidney disease, female sex, diabetes mellitus, COPD，HF is the predictor of early readmission. Initially, chronic kidney disease is commonly correlation with dyslipidemia, diabetes, and hypertension which result in atherosclerosis and endothelial dysfunction, so it is considered as intensively independent risk factor of patients diagnosed AMI.^26^ The second risk factor is gender. Indeed, past research has shown that women were at higher risk of post‐AMI 30‐day readmission than men, especially younger women.^27^ Females probable have different pathophysiological and clinical characteristics from men,^19^ for example，women often have experience chest pain or myocardial ischemia rather than coronary artery obstruction.^27^ Then, the risk factors for AMI in patients with diabetes mellitus were more than twice as high as in patients without diabetic of AMI.^28^ The AMI people with renal insufficiency and diabetes mellitus are relevant to major adverse cardiac events and risk of unfavorable prognosis, which can offer worthwhile information for early risk stratification.^29^ Lastly, Hawkins et al^30^ revealed that COPD increased mortality and non‐fatal clinical events in patients with acute myocardial infarction. Other risk factors of early readmission patients with AMI are HF, peripheral vascular disease, and cardiac arrhythmia. Studies indicated that revascularization have declined odds compared with those whose did not received interventional procedure for 30‐day readmission rate after myocardial infarction, especially in patients who have underwent PCI.^7^, ^12^ Similarity, previous studies also indicated that only 8.0% of patients were readmitted in the 30 days after a PCI.^31^ However, other studies have shown that the 30‐day readmission rate after myocardial infarction is as high as 17.5%,^32^ which is higher than our meta‐analysis. Whether PCI can reduce the readmission rate by 30 days is the subject of ongoing debate. But it is established that PCI can decrease long‐term mortality and morbidity and improve prognosis.

Currently, a good deal of articles developed risk prediction models for hospital readmission of AMI. However, Smith et al^33^ indicated there is no real‐time operational information model that can identify and stratify the risk of AMI patients before the hospital discharge of the AMI patients through study of 11 articles in 16 AMI risk prediction models. Among the 15 risk prediction models for 30 day readmission had poor discrimination with a median C‐statistic of 0.65, and were of uncertain universality due to methodological quality limitations.^33^ Additional review suggested that there is no effective model to measure the re‐admission rate of hospital or to establish re‐admission risk model for individual patients.^34^ Most studies which lacked of models validation or being a single‐center study were evaluated low to moderate quality. In order to avoid the limitation of the individual, we would use the method of pooled multiple studies to provide consistently identified variables.

This review was to summarize the potential risk factors and reasons by useful available literature to generate strategies to reduce 30‐day readmissions. Early outpatient physical follow‐up has been reviewed as an effective strategy to prevent readmission. However, in American hospitals with higher early follow‐up rates after AMI do not effectively reduce the 30‐day admission rate，so it is necessary to adopt other targeted strategies besides early doctor follow‐up to reduce the readmission rate of this population.^35^ In particular, we should take into account research on predictors of readmission, risk stratification, interventions, risk‐standardized model to reduce 30‐day readmissions.

## 5 LIMITATIONS

This review of results has certain several limitations. First, because of different articles have various classifications and grouping of causes and risk factors, there are inconsistent definitions for the studied variables, which make combining them for meta‐analysis difficult. For example, different age groups were studied and used to make it impossible to merge and analyze data. Similarly, many risk factors and causes of readmission were unclearly defined, which potentially lead to overlap or deletion of data. Second, the substantial heterogeneity between studies may be come from age, data source, study period, diagnose, study population as well as study designs. Due to the limitations of the data, we could not conduct subgroup analysis for each variable. As mentioned above, the majority of studies did not offer complete data, which some studies only probed readmission rates and did not include analyses of risk factors and causes.

## 6 CONCLUSIONS

In conclusion, the 30‐day readmission rate post‐AMI ranged from 11% to 14% and can mainly come from cardiovascular and non‐cardiovascular reasons. ACS, angina and ischemic heart disease, heart failure, and acute myocardial infarction were the principal cardiovascular reasons of 30‐day readmission. Meanwhile, non‐specific chest pain was regarded as the significant cause among non‐cardiovascular reasons. The common comorbidity such as kidney disease, HF, COPD, and diabetes mellitus were significant risk factors for 30‐day readmission. Therefore, our finding can help develop targeted strategies and prediction model to lower readmission rates according to the underlying risk factors and reasons of the 30‐day readmission after AMI.

## CONFLICT OF INTEREST

The authors declare no potential conflict of interests.

## Supporting information


**APPENDIX S1** The detailed search strategy

**APPENDIX S2** Quality assessment using

**APPENDIX S3** Funnel plots of 30‐day readmission rate after acute myocardial infarction

**APPENDIX S4** Subgroup analysis for 30‐day readmission after acute myocardial infarction

**APPENDIX S5** Influence analysis of 30‐day readmission rate after acute myocardial infarction
