# Supplementary material for: The prevalence of 30‐day readmission after acute myocardial infarction: A systematic review and meta‐analysis
Source: Clin Cardiol. 2019 Aug 12;42(10):889–98. doi: 10.1002/clc.23238 (PMC6788479; doi:10.1002/clc.23238)
Supplement: Supplementary file 1 — APPENDIX S1 The detailed search strategy [file CLC-42-889-s001.docx]

| **Supplementary appendix 1. The detailed search strategy** | | | |
| --- | --- | --- | --- |
| **Source: PubMed; Searched on: May 26 2019; Results: 138** | | | |
| Search | | Query | Items found |
| #1 | | Search readmission*[Title] | 6,125 |
| #2 | | Search re-admission*[Title] | 164 |
| #3 | | Search rehospitalization*[Title] | 736 |
| #4 | | Search re-hospitalization*[Title] | 89 |
| #5 | | Search reattendance*[Title] | 33 |
| #6 | | Search re-attendance*[Title] | 19 |
| #7 | | Search readmittance*[Title] | 2 |
| #8 | | Search re-admittance* [Title] | 0 |
| #9 | | Search (#1 OR #2 OR #3 OR #4 OR #5 OR #6 OR #7 OR #8 ) | 7,158 |
| #10 | | Search “acute myocardial infarction ”[Title] | 28,987 |
| #11 | | Search “AMI”[Title] | 961 |
| #12 | | Search “myocardial infarction”[Title] | 72,761 |
| #13 | | Search “MI”[Title] | 1,844 |
| #14 | | Search “Non-ST-Segment Elevation Myocardial Infarction”[Title] | 478 |
| #15 | | Search “NSTEMI”[Title] | 172 |
| #16 | | Search “ST-elevation myocardial infarction”[Title] | 3,608 |
| #17 | | Search “STEMI”[Title] | 1,415 |
| #18 | | Search (#10 OR #11 OR #12 OR #13 OR #14 OR #15 OR #16 OR #17) | 76318 |
| #19 | | Search (#9 AND # 18) | 138 |
|  | |  |  |
| **Source: Embase; Searched on: May 26 2019; Results: 269** | | | |
| Search | | Query | Items found |
| #1 | | readmission*:ti | 10,325 |
| #2 | | re-admission*:ti | 363 |
| #3 | | rehospitalization*:ti | 1,115 |
| #4 | | re-hospitalization*:ti | 242 |
| #5 | | reattendance*:ti | 48 |
| #6 | | re-attendance* :ti | 31 |
| #7 | | readmittance*:ti | 3 |
| #8 | | re-admittance*:ti | 3 |
| #9 | | #1 OR #2 OR #3 OR #4 OR #5 OR #6 OR #7 OR #8 | 12,114 |
| #10 | | “acute myocardial infarction”:ti | 41,097 |
| #11 | | “AMI”:ti | 1,813 |
| #12 | | “myocardial infarction”:ti | 103,953 |
| #13 | | “MI”:ti | 3,363 |
| #14 | | “Non ST Segment Elevation Myocardial Infarction”:ti | 710 |
| #15 | | “NSTEMI”:ti | 528 |
| #16 | | “ST-elevation myocardial infarction”:ti | 6,735 |
| #17 | | “STEMI”:ti | 4,381 |
| #18 | | #10 OR #11 OR #12 OR #13 OR #14 OR #15 OR #16 OR #17 | 112,002 |
| #19 | | #9 AND #18 | 269 |
|  | |  |  |
| **Source: The Cochrane Library; Searched on: May 26 2019; Results: 697** | | | |
| Search | Query | | Items found |
| #1 | readmission*:ti,ab,kw | | 5,833 |
| #2 | re-admission*:ti,ab,kw | | 5,831 |
| #3 | rehospitalization*:ti,ab,kw | | 1,693 |
| #4 | re-hospitalization*:ti,ab,kw | | 1,693 |
| #5 | reattendance*:ti,ab,kw | | 70 |
| #6 | re-attendance*:ti,ab,kw | | 70 |
| #7 | readmittance*:ti,ab,kw | | 24 |
| #8 | re-admittance*:ti,ab,kw | | 24 |
| #9 | #1 OR #2 OR #3 OR #4 OR #5 OR #6 OR #7 OR #8 | | 6,848 |
| #10 | “acute myocardial infarction”:ti,ab,kw | | 8,290 |
| #11 | “AMI”:ti,ab,kw | | 3,013 |
| #12 | “myocardial infarction”:ti,ab,kw | | 27,576 |
| #13 | “MI”:ti,ab,kw | | 9,222 |
| #14 | “Non ST Segment Elevation Myocardial Infarction”:ti,ab,kw | | 733 |
| #15 | “NSTEMI”:ti,ab,kw | | 474 |
| #16 | “ST-elevation myocardial infarction”:ti,ab,kw | | 1,929 |
| #17 | “STEMI”:ti,ab,kw | | 2,877 |
| #18 | #10 OR #11 OR #12 OR #13 OR #14 OR #15 OR #16 OR #17 | | 32,845 |
| #19 | #9 AND #18 | | 697 |
