# Supplementary material for: The prevalence of 30‐day readmission after acute myocardial infarction: A systematic review and meta‐analysis
Source: Clin Cardiol. 2019 Aug 12;42(10):889–98. doi: 10.1002/clc.23238 (PMC6788479; doi:10.1002/clc.23238)
Supplement: Supplementary file 2 — APPENDIX S2 Quality assessment using [file CLC-42-889-s002.docx]

| Appendix 2. Quality assessment using | | | | | | | | | |
| --- | --- | --- | --- | --- | --- | --- | --- | --- | --- |
| Author(year) | Sample size (n) | Sample design | Sampling frame | Study and setting | Measures | Unbiased assessors | Response rate and refusers | Prevalence rates | Total score |
| Li et al.2019 | * | * | * | * | * | — | * | — | 6 |
| Zabawa et al.2018 | * | — | * | * | * | — | * | — | 5 |
| Nguyen et al.2018 | * | * | * | * | * | — | * | — | 6 |
| Kim et al.2018 | * | * | * | * | * | — | * | — | 6 |
| Rodriguez-Padial et al. 2018 | * | * | * | * | * | — | * | — | 6 |
| Kwok et al.2017 | * | * | * | * | * | — | * | — | 6 |
| Khera et al.2017 | * | * | * | * | * | — | * | — | 6 |
| Tisminetzky et al.2016 | * | * | * | * | * | — | * | — | 6 |
| Dreyer et al.2015 | * | — | * | * | * | — | * | — | 5 |
| Chen et al.2015 | * | * | * | * | * | — | * | — | 6 |
| Ranasinghe et al.2014 | * | * | * | * | * | — | * | — | 6 |
| Ben-Assa E et al.2014 | * | * | — | * | — | — | * | — | 4 |
| Brown et al.2013 | * | * | * | * | * | — | * | — | 6 |
| Dunlay et al.2012 | * | * | * | * | * | — | * | — | 6 |
