# Supplementary figures and images for: The prevalence of 30‐day readmission after acute myocardial infarction: A systematic review and meta‐analysis
Source: Clin Cardiol. 2019 Aug 12;42(10):889–98. doi: 10.1002/clc.23238 (PMC6788479; doi:10.1002/clc.23238)

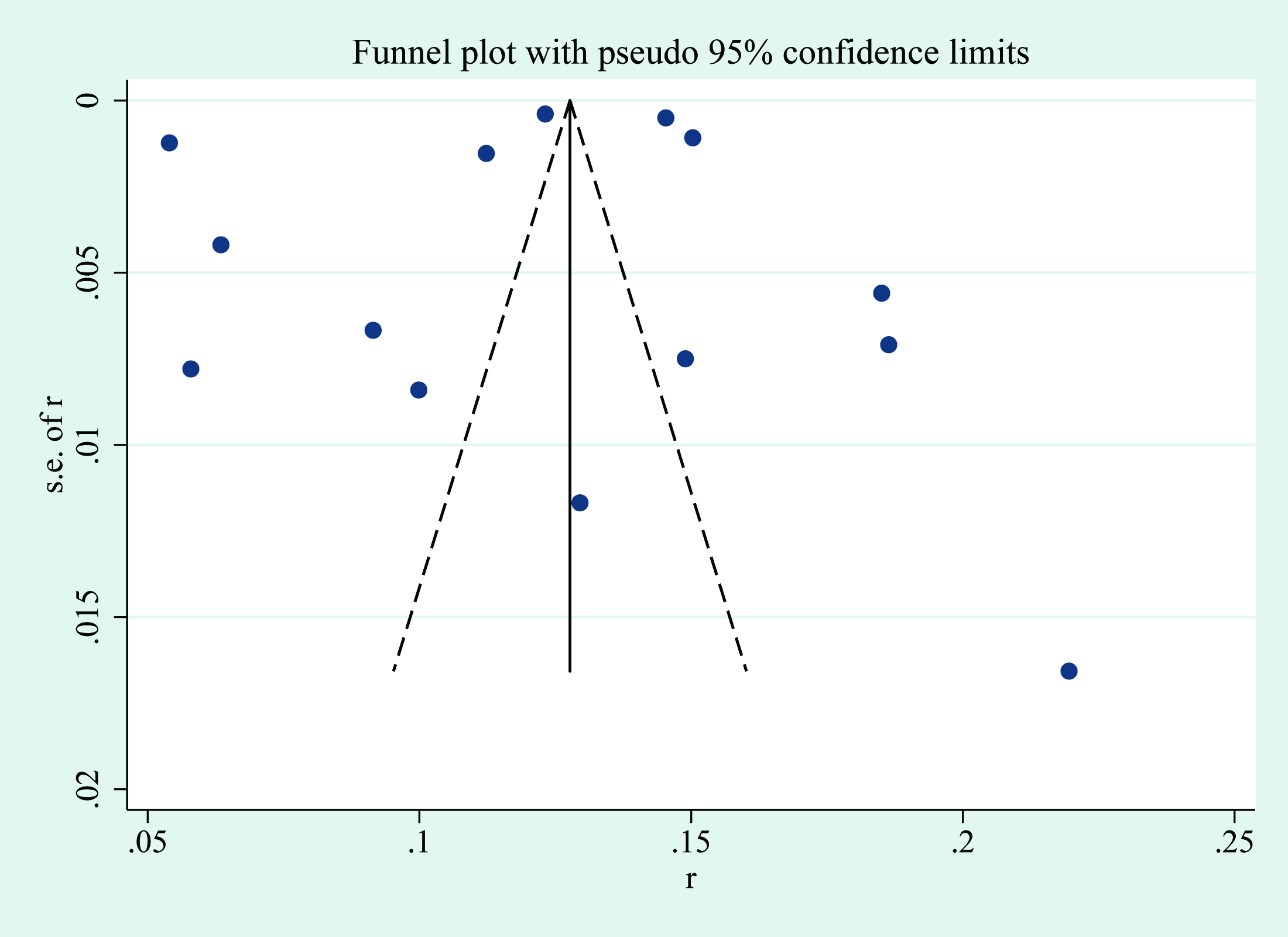

Supplement: Supplementary file 3 — APPENDIX S3 Funnel plots of 30‐day readmission rate after acute myocardial infarction [file CLC-42-889-s003.tif]

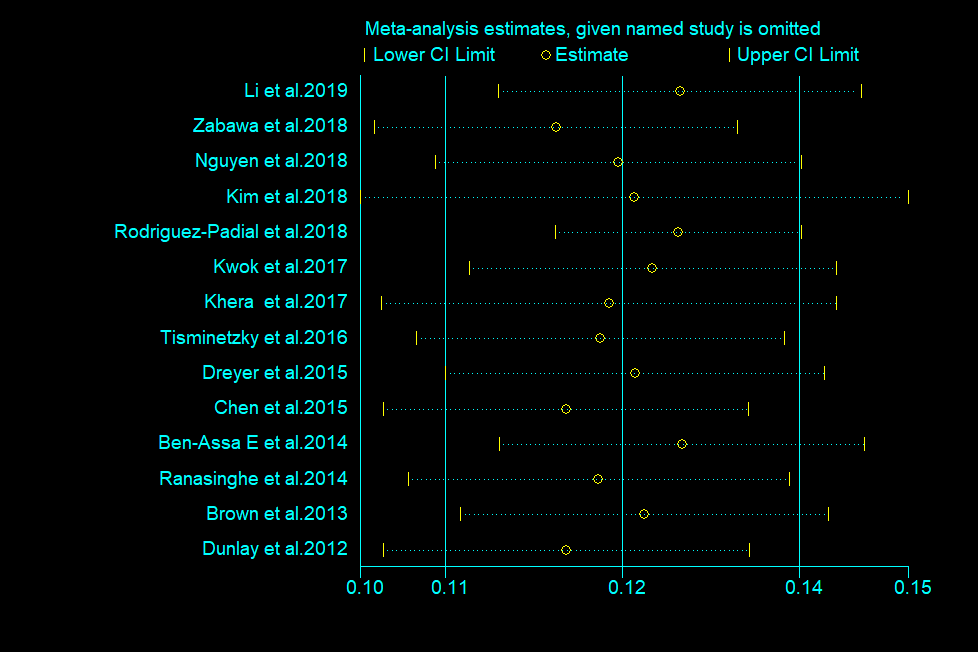

Supplement: Supplementary file 5 — APPENDIX S5 Influence analysis of 30‐day readmission rate after acute myocardial infarction [file CLC-42-889-s005.tif]
