# Supplementary material for: The prevalence of 30‐day readmission after acute myocardial infarction: A systematic review and meta‐analysis
Source: Clin Cardiol. 2019 Aug 12;42(10):889–98. doi: 10.1002/clc.23238 (PMC6788479; doi:10.1002/clc.23238)
Supplement: Supplementary file 4 — APPENDIX S4 Subgroup analysis for 30‐day readmission after acute myocardial infarction [file CLC-42-889-s004.docx]

| Appendix 4：Subgroup analysis for 30-dayreadmission after AMI | | | | | |
| --- | --- | --- | --- | --- | --- |
| Items | NO. of studies | | | Pooled rate with 95%CI | I^2^ (%) |
| Pooled results |  | | |  |  |
| Region |  | | |  |  |
| Non-America | 4 | | | 0.10（0.07,0.13） | 97.7 |
| America | 10 | | | 0.13（0.12,0.15） | 99.8 |
| Sample size | | | | | |
| <1000 | 3 | | | 0.13(0.05,0.22) | 97.7 |
| 1000-10000 | 6 | | | 0.13(0.08,0.18) | 98.9 |
| >10000 | 5 | | | 0.12(0.09,0.14) | 99.9 |
| Quality of included studies | | | |  |  |
| ≥ 6 | 11 | | | 0.12(0.11,0.14) | 99.8 |
| < 6 | 3 | | | 0.13(0.07,0.18) | 97.8 |
| Definition of 30-day readmission | | | | | |
| Unplanned | | 4 | 0.11（0.06,0.16） | | 99.4 |
| Readmission | | 10 | 0.13 (0.11,0.15) | | 99.8 |
